# Endoluminal photodynamic therapy with a photoreactive stent‐based catheter system to treat malignant colorectal obstruction

**DOI:** 10.1002/btm2.10732

**Published:** 2024-11-23

**Authors:** Seung Jin Eo, Dae Sung Ryu, Hyeonseung Lee, Ji Won Kim, Song Hee Kim, Jin Hee Noh, Yuri Kim, Seokin Kang, Kun Na, Jung‐Hoon Park, Do Hoon Kim

**Affiliations:** ^1^ Biomedical Engineering Research Center, Asan Institute for Life Sciences Asan Medical Center Seoul Republic of Korea; ^2^ Department of Convergence Medicine, Asan Medical Center University of Ulsan College of Medicine Seoul Republic of Korea; ^3^ Department of Gastroenterology, Asan Medical Center University of Ulsan College of Medicine Seoul Republic of Korea; ^4^ Department of Biotechnology, Department of Biomedical‐Chemical Engineering The Catholic University of Korea Bucheon‐si Gyeonggi‐do Republic of Korea; ^5^ Department of Internal Medicine, Ilsan Paik Hospital Inje University College of Medicine Goyang‐si Gyeonggi‐do Republic of Korea

**Keywords:** ablation, catheter, colorectal cancer, photodynamic therapy, self‐expandable metallic stent

## Abstract

Photodynamic therapy (PDT) using photosensitizer (PS)‐embedded silicone membrane‐covered self‐expandable metallic stents (SEMSs) can function in palliative therapeutic option for malignant gastrointestinal tract obstruction. However, stent‐related complications should be considered, and accurate delivery of light sources is technically difficult. Here, a Chlorin e6 (Ce6)‐an embedded stent‐based catheter is developed to improve its therapeutic efficacy and safety. PDT using Ce6‐embedded stent successfully induced cell death of colorectal cancer cell line. PDT‐treated liver tissues showed an increase in ablation depth in proportion to irradiation energy, and 600 J/cm^2^ demonstrates an even and sufficient ablation depth. Endoluminal PDT using the Ce6‐embedded stent‐based catheter was technically successful in a rat colon model without procedure‐related complications such as colonic perforation or stricture formation. The results in colonoscopy, colonography, and histological examination, along with statistical analysis, suggest that a novel PDT modality using a Ce6‐embedded stent‐based catheter was safely conducted and demonstrated apoptotic cell death at 12 h after PDT, and it gradually recovered from 2 to 4 weeks. Thus, the PDT using the Ce6‐embedded stent‐based catheter may represent a promising new approach for the treatment of malignant colorectal obstruction.

**Translational Impact Statement**

PDT using PS‐embedded membrane‐covered SEMS emerged as a palliative therapeutic option for malignant GI obstruction. However, stent‐related complications must be considered, and accurate delivery of light sources is technically challenging. We introduce a localized PDT method using a PS‐embedded stent‐based catheter system. Our results reveal apoptotic cell death 12 h after PDT without procedure‐related complications in a rat colon model. The findings can provide valuable insights for the translation of localized PDT as a routine clinical alternative to the treatment of malignant GI obstruction.

## 1 INTRODUCTION

A self‐expandable metallic stent (SEMS) is used as a palliative option in unresectable malignant colorectal obstruction because it helps avoid a palliative colostomy and shorten the hospital stay,^1^, ^2^, ^3^ or as a pre‐operative bridge to surgical resection in patients with primary colonic tumors.^4^ However, bare SEMSs are associated with tumor in‐growth rates of 3%–46% through the wire mesh, and covered SEMSs are associated with significant stent migration rates of 9%–10%.^5^, ^6^, ^7^, ^8^, ^9^ Recently, stent‐based radiofrequency ablation (RFA) and irreversible electroporation (IRE) have been investigated for endoluminal ablation in the local treatment of malignant obstruction in non‐vascular luminal organs, such as the esophagus, bile duct, and gastric outlet.^10^, ^11^, ^12^, ^13^ Both localized ablation methods using self‐expandable stent‐based electrodes are technically feasible and effective in evenly inducing cell death in endoluminal tumors. However, RFA and IRE procedures for non‐vascular luminal organs often demonstrated severe complications, including perforation, fistula, and bleeding.^14^, ^15^ Furthermore, in the case of the colorectum with its relatively thin wall,^16^ endoluminal RFA and IRE are challenging and risky procedures. Therefore, alternative ablation modalities for local treatment of malignant colorectal obstruction need to be explored.

Photodynamic therapy (PDT) is a promising alternative ablation method based on chemical damage induced by photosensitized reactions.^17^ PDT is the dynamic interaction between a photosensitizer (PS), light with a specific wavelength, and molecular oxygen, promoting the selective destruction of the target tissue.^18^ Upon irradiation with light of the appropriate wavelength, the PS generates highly reactive oxygen species (ROS), which can directly destroy tumor cells by inducing apoptosis and necrosis.^19^ However, the critical drawbacks of most PSs that limit their clinical applications in PDT include their poor water solubility and short blood circulation time after following intravenous injection with subsequent insufficient accumulation in tumor tissues.^20^, ^21^ To overcome these problems, Bae et al. suggested that a PS‐embedded membrane‐covered SEMS could be a palliative therapeutic option for malignant biliary obstruction and allows repeatable PDT directly to the tumoral bile duct without the systemic injection of a PS.^22^ However, since the stent should be placed into the bile duct, stent‐related complications must be considered.^23^, ^24^ Additionally, the placement of the flexible fiber in the exact center position of the luminal space is technically challenging. Recently, Chlorin‐e6 (Ce6), which has high ROS generation ability, has been widely investigated as a photosensitizer,^25^, ^26^, ^27^ and it was successfully embedded into a silicone membrane.^28^, ^29^ A Ce6‐embedded silicone‐covered device to treat obesity has been demonstrated to actively generate ROS following irradiation with light, resulting in cell damage in tissues in contact with the Ce6‐embedded silicone membrane and induced apoptosis with inflammatory reactions. Meanwhile, PDT‐induced ablation depth is relatively restricted because the depth penetration of a near‐infrared laser is approximately 5–6 mm. However, limited depth penetration of PDT may be a safer ablation treatment over RFA or IRE in the colorectum.

**FIGURE 1:**
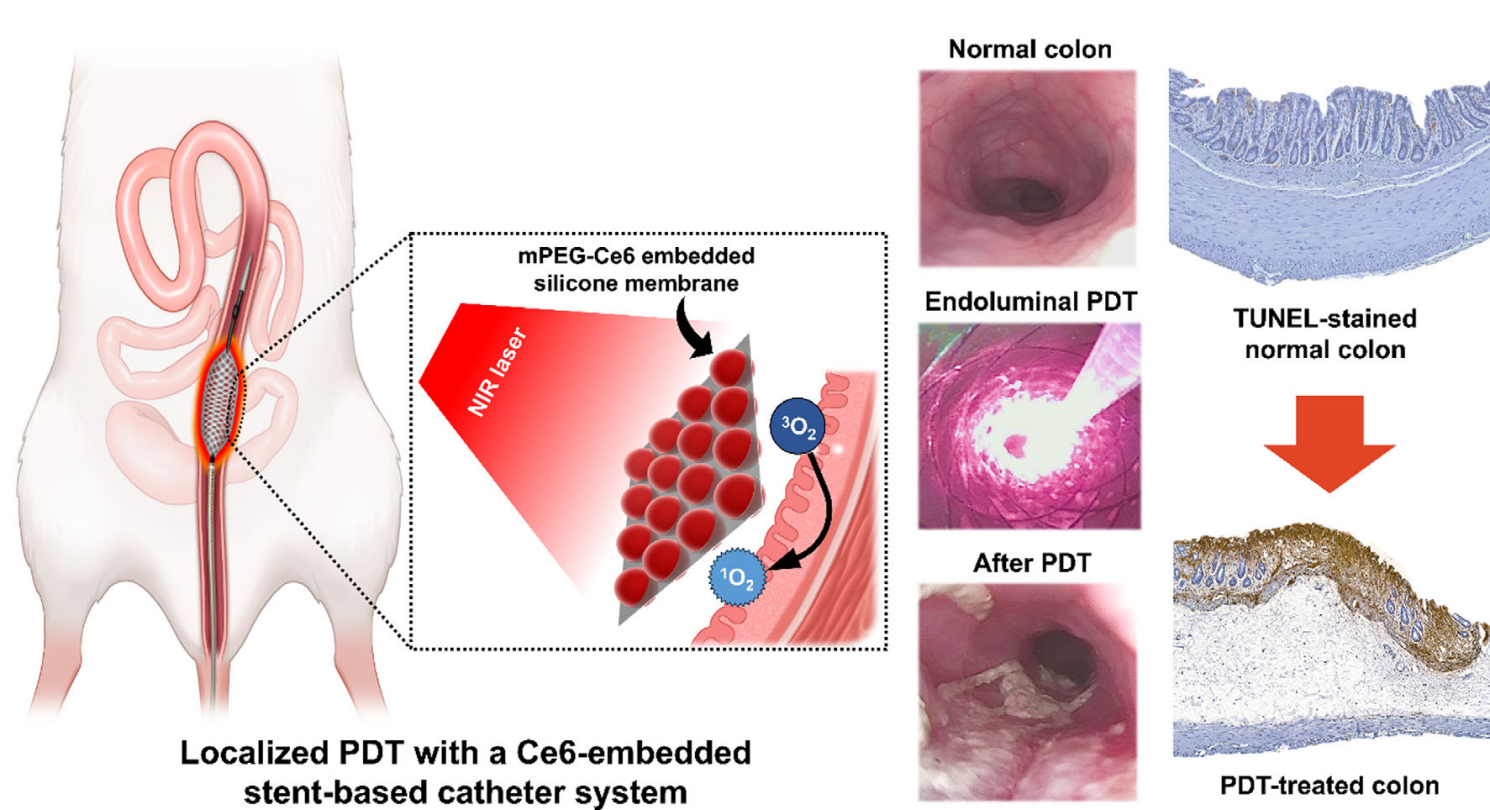
Schematic illustration of localized PDT using a Ce6‐embedded stent‐based catheter system in the rat colon, and the representative colonoscopic images were obtained during the procedures with TUNEL‐stained normal and PDT‐treated colon microscopic images demonstrating the therapeutic effects. Ce6, Chlorin‐e6; PDT, photodynamic therapy; TUNEL, terminal deoxynucleotidyl transferase‐mediated dUTP.

A Ce6‐embedded stent‐based catheter system was designed and developed for the local treatment of malignant colorectal obstructions (Figure 1). The Ce6‐embedded stent was loaded into the catheter system, and both ends of the stent were fixed to enable recapture and removal. The cylindrical fiber can be easily inserted through the inner tube and positioned at the exact center of the Ce6‐embedded stent to perform accurate and effective PDT. This novel stent‐based catheter system with a recapturable Ce6‐embedded stent may generate cytotoxic singlet oxygen under catheter‐based light activation while avoiding stent‐ and PS‐related complications. Fabrication of methoxy polyethylene glycol amine (mPEG)‐Ce6‐embedded stent was confirmed by Fourier transform infrared spectroscopy (FT‐IR) and singlet oxygen sensor green (SOSG) fluorescence. To investigate the suitability of the Ce6‐embedded stent with the push‐pull design of a catheter, a Ce6‐embedded stent‐based catheter was characterized before and after deployment. Mechanical properties of the radial and axial force of the stent portion were measured and quantified. In vitro cell study with Ce6‐embedded pieces was performed to evaluate the cytotoxicity and phototoxicity in colon cancer cells. The dose‐range study with the Ce6‐embedded stent was conducted to determine the optimal irradiation energy for even ablation in the colon. Finally, PDT using Ce6‐embedded stent‐based catheter system was conducted in a rat colon model. Additionally, colonoscopy, colonography, and histological examination were conducted along with the statistical analysis, which demonstrated the efficacy and safety of the PDT using the Ce6‐embedded stent‐based catheter system. Thus, PDT using the Ce6‐embedded stent‐based catheter system is a promising new approach for the treatment of malignant colorectal obstruction.

## 2 RESULTS AND DISCUSSION

### 2.1 Characterization of mPEG‐Ce6‐embedded stent‐based catheter system

The Ce6‐embedded stent‐based catheter system was successfully fabricated (Figure S1). In previous studies, a PS‐embedded stent was placed at the target site, and the optic fiber was subsequently advanced under endoscopic guidance. However, positioning the laser irradiation at the center of the stent was technically difficult, and controlling the fiber within the exact center position of the luminal space is almost impossible. In our novel catheter system, the optic fiber can be easily positioned in the exact middle portion of the PS‐embedded stent through the inner tube of the catheter. By placing the fiber in the precise center position, energy is evenly delivered to all the PS‐embedded stented areas. Furthermore, placed PS‐embedded stent can be safely removed immediately after PDT without stent‐related complications. Therefore, PDT using the PS‐embedded stent‐based catheter system may be a promising, accurate, and convenient new option of PDT procedure.

**FIGURE 2:**
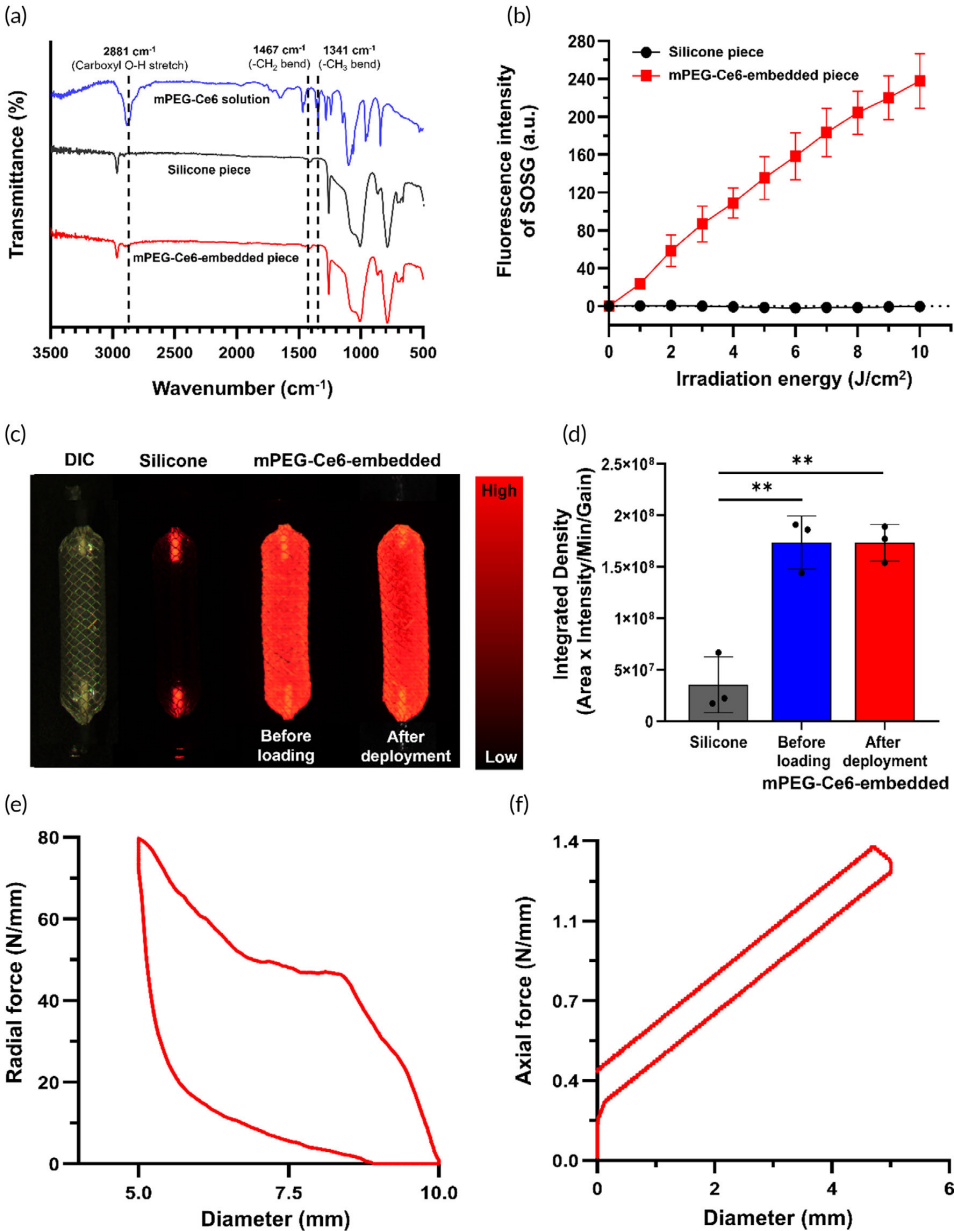
Characterization of the mPEG‐Ce6‐embedded stent‐based catheter system. (a) FT‐IR spectra of mPEG‐Ce6 solution, silicone, and mPEG‐Ce6‐embedded samples. (b) SOSG fluorescent intensity of silicone and mPEG‐Ce6‐embedded samples in aqueous conditions with 0.1% DMSO under laser irradiation (total 10 J/cm^2^; 20 mW/cm^2^, 20 s, 25 times of measurement). (c) Fluorescence images obtained from the silicone stent, before loading and after deployment of the mPEG‐Ce6‐embedded stent. (d) Graph showing the integrated density in Figure 2c. Graphs showing (e) radial (79.72 N/mm) and (f) axial (1.37 N/mm) force of the mPEG‐Ce6‐embedded stent‐based catheter system. Data are presented as mean ± standard deviation. ** *p* < 0.01. Ce6, Chlorin‐e6; FT‐IR, Fourier Transform‐Infrared Spectroscopy; mPEG, methoxypolyethylene glycol amine; SOSG, singlet oxygen sensor green.

Characteristics of mPEG‐Ce6‐embedded silicone membrane were described in a previous study.^28^, ^29^ After dispersing mPEG‐Ce6 in a silicone solution and fabricating a film, the presence of mPEG‐Ce6 within the silicon film was confirmed by FT‐IR. Distinctive peaks at both 1007 and 1261 cm^−1^, representing the asymmetric vibrations of Si—O—Si, along with peaks at 465 and 787 cm^−1^, which correlated with the bending vibrations of Si—O—Si, were observed in both the silicon and mPEG‐Ce6 film.^30^ In contrast, the mPEG‐Ce6 film demonstrated new peaks at 1341, 1467, and 2881 cm^−1^, which corresponded to the —CH_3_ bend from Ce6 and mPEG, —CH_2_ bend from mPEG, and Carboxyl O—H stretch from Ce6, respectively (Figure 2a). Through this spectrum analysis, it was determined that mPEG‐Ce6 was indeed embedded in the silicon film. As determined by SOSG fluorescence, the mPEG‐Ce6‐embedded piece successfully generated singlet oxygen and exhibited higher fluorescence intensity of SOSG compared to silicone piece (Figure 2b). The homogeneity of the mPEG‐Ce6 silicone membrane of stent‐based catheter was identified using fluorescence images, which revealed significantly more homogenous and stronger photo‐fluorescence intensity compared with that of only silicone stent (*p* < 0.01, two‐sample *t*‐test). To perform the localized PDT procedure, the mPEG‐Ce6‐embedded stent should be loaded into the delivery system and then expanded at the target site.^31^ During this process, the coating layer can be damaged due to friction with the delivery system.^32^ To investigate the stabilization of the mPEG‐Ce6‐embedded silicone membrane onto the surface of the stent, fluorescence intensity was evaluated before loading and after deployment of the mPEG‐Ce6‐embedded stent to evaluate the fluorescence intensity changes (Figure 2c,d). No significant difference was observed between the two samples. The mPEG‐Ce6‐embedded stent proved suitable for a push‐pull catheter design. Meanwhile, a Ce6 with a small molecule may be easily released from the silicone membrane. PEGylation increased the molecular weight and prevented Ce6 elution from the membrane. The residual Ce6 level was more than 98% at 9 days in a previous study.^27^ Therefore, the increased molecular weight successfully inhibited unexpected toxicity caused by released PS from the membrane.

### 2.2 Mechanical properties of mPEG‐Ce6‐embedded stent‐based catheter system

The radial and axial maximum forces of expanded stent‐based catheter system were 79.72 and 1.37 N/mm, respectively (Figure 2e,f). Commercially available non‐vascular covered SEMSs have radial force in the range of 40–83 N and axial force in the range of 0.4–2.7 N.^33^ Compared with the previous data, the mechanical properties of the mPEG‐Ce6‐embedded stent‐based catheter system showed relatively high radial and low axial forces. High radial force contributes to fully contact with the colonic wall for enhanced PDT effects. The low axial force provides less traumatic injuries to the tissues and is more pliable to the irregular or curved GI tract. Thus, the stent portion of the mPEG‐Ce6‐embedded stent‐based catheter system could provide safely sufficient luminal patency while in full contact with endoluminal tissue to perform effective PDT procedure.

### 2.3 Cytotoxicity and phototoxicity of the Ce6‐embedded stent in colon cancer cells

**FIGURE 3:**
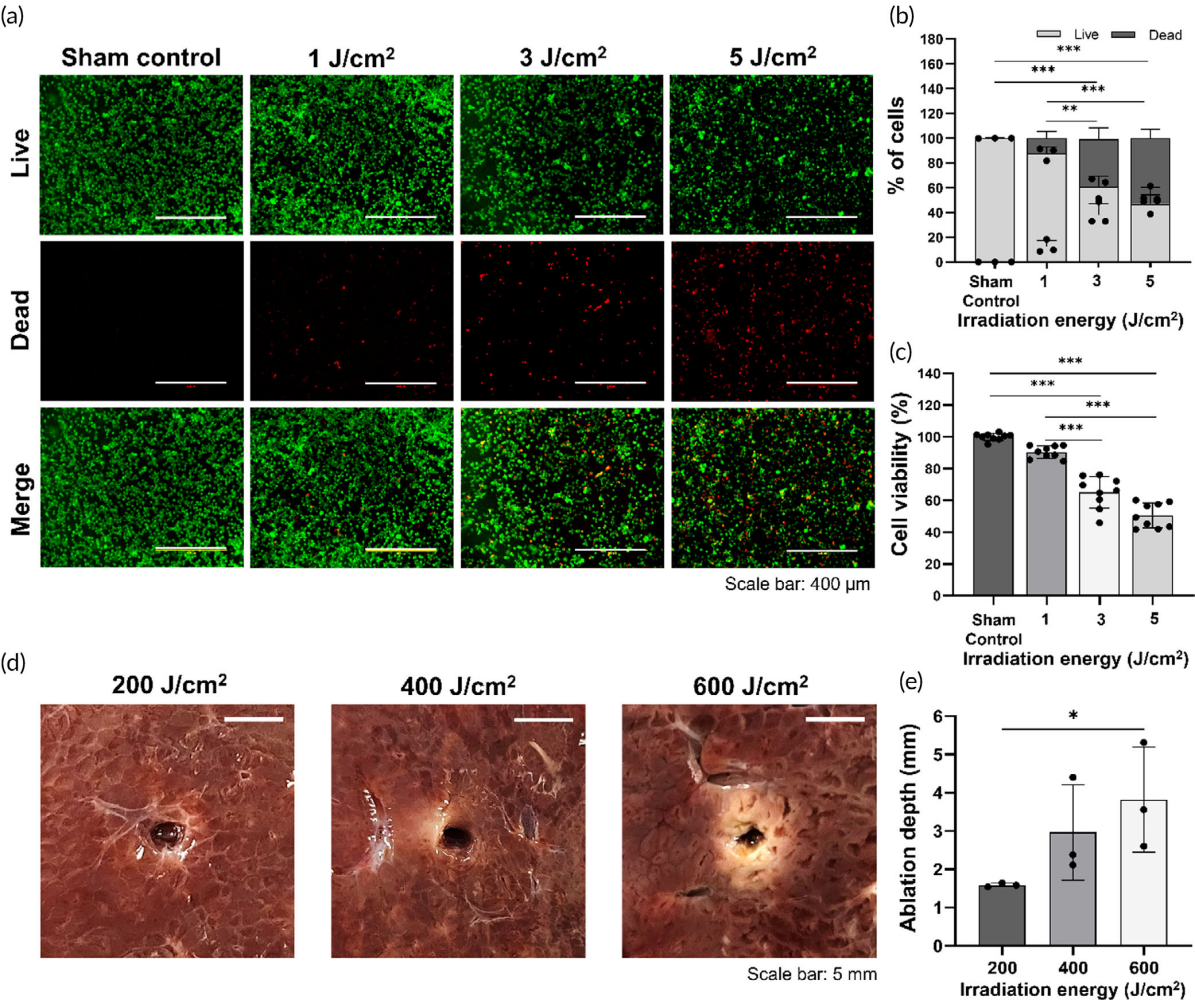
In vitro phototoxicity and cytotoxicity in colon cancer cells and dose‐range study in the porcine liver tissues. (a) In vitro live and dead cell assay of CT‐26 cells using PDT under various irradiation energy. Live and dead cells were stained with Calcein AM (live; green color) and EthD‐1 (dead; red color), respectively (scale bars: 400 μm). (b) Graph showing the quantitative results of the live and dead cell assay findings. (c) In vitro cell viability of CT‐26 cells using PDT under various irradiation energy. (d) Representative photographs of TTC‐stained liver tissues 12 h after the PDT with 200, 400, and 600 J/cm^2^ (scale bars: 5 mm). Ablation depths proportionally increased with an increase in irradiation energy. (e) Graph showing the results of ablation depth under various irradiation energy. Data are presented as mean ± standard deviation. * *p* < 0.05, ** *p* < 0.01, *** *p* < 0.001. Ce6, Chlorin‐e6; PDT, photodynamic therapy; TTC, 3,5‐triphenyltetrazolium chloride.

The Ce6‐embedded pieces were introduced to CT‐26 cell lines and activated with laser irradiation (670 nm; 1, 3, or 5 J/cm^2^) to evaluate the cytotoxicity and phototoxicity of the Ce6‐embedded silicone membrane using live/dead and CCK‐8 assays (Figure S2). In in vitro cell studies for the localized PDT, light doses of 1–5 J/cm^2^ are frequently used to confirm the therapeutic effects, and the energy dose tends to increase as the research progresses to the ex vivo or in vivo study (mouse, rat, and porcine).^22^, ^34^, ^35^ However, the limited number of research on localized PDT was not enough to establish a correlation between in vitro and ex vivo. The live/dead assay demonstrated that the rates of dead cells were significantly increased in proportion with the irradiation energy dose (Figure 3a). The mean (±standard deviation [SD]) proportion of dead cells was 0.01 ± 0.06% in the sham control group, 12.29 ± 4.30% at 1 J/cm^2^, 38.12 ± 7.46% at 3 J/cm^2^, and 53.07 ± 5.86% at 5 J/cm^2^, respectively (all variables, *p* < 0.001, one‐way ANOVA) (Figure 3b). The mean proportion of dead cells in the 3 and 5 J/cm^2^ were significantly higher than those that in the sham control and 1 J/cm^2^ (all variables except for 3 J/cm^2^ vs. 1 J/cm^2^, *p* < 0.001; 3 J/cm^2^ vs. 1 J/cm^2^, *p* < 0.01, one‐way ANOVA). In the CCK‐8 assay, the mean (±SD) cell viability was 99.99% ± 2.11% in the sham control, 90.40% ± 3.65% with 1 J/cm^2^, 65.02% ± 9.46% with 3 J/cm^2^ and 50.55% ± 7.34% with 5 J/cm^2^, respectively (all variables, *p* < 0.001, one‐way ANOVA). The mean cell viabilities in the 3 and 5 J/cm^2^ were significantly higher than those that in the sham control and 1 J/cm^2^ (all variables, *p* < 0.001, one‐way ANOVA). (Figure 3c). PDT is a highly complex treatment with several parameters affecting its efficacy (e.g., type of PS, PS dose, light source, light fluence, irradiation distance).^36^, ^37^, ^38^ A previous study with PDT using a PS‐embedded membrane demonstrated that the proportion of dead cells increased significantly with increasing laser intensity.^29^, ^35^ Consequently, we selected irradiation energy as the main parameter and similar results were observed in our findings of the in vitro cell study. Furthermore, our novel PDT system with a stent‐based catheter helps maintain and control various PDT parameters, including irradiation distance, light source delivery, and PS dose. Therefore, optimized irradiation energy using the PS‐embedded stent‐based catheter system can contribute to consistent results of the PDT‐induced therapeutic effects.

### 2.4 Dose‐range study using 2,3,5‐triphenyl tetrazolium chloride‐stained porcine liver tissue

TTC staining is used to identify the area of cell death in PDT‐treated liver tissues (Figure S3). It is used as a redox indicator to differentiate between metabolically active and inactive tissues. Therefore, it is often used to distinguish between areas of live and dead cells in PDT studies.^39^, ^40^, ^41^ PDT‐treated and TTC‐stained liver tissue samples are shown in Figure 3d. The mean (±SD) ablation depth was 1.58 ± 0.05 mm with 200 J/cm^2^, 2.96 ± 1.25 mm with 400 J/cm^2^, and 3.89 ± 1.28 mm with 600 J/cm^2^, respectively (Figure 3e). The ablation depth was significantly higher with 600 J/cm^2^ than with 200 J/cm^2^ (*p* < 0.05, two‐sample *t‐test*). The ablation depth also increased proportionally with the irradiation energy, and the ablation area of the TTC‐stained liver was clearly identified. The PDT‐treated livers at 400 J/cm^2^ showed irregular ablation areas compared with 600 J/cm^2^. The NIR power intensity with a wavelength of 670 nm are frequently used in clinical practice for PDT procedure with the oncological ranges of 100–600 J/cm^2^.^42^ In our study, the PDT‐treated liver at 600 J/cm^2^ demonstrated sufficient ablation depth and even ablation patterns of the Ce6‐embedded stent‐based catheter. Our ex vivo study demonstrated that PDT using the Ce6‐embedded stent could induce sufficient ablation for localized endoluminal treatment for malignant strictures. Additionally, the ablation may be easily adjusted according to the irradiation energy dose. Mlkvy et al. reported on the effective treatment depth ranging between 5 and 18 mm in depth of necrosis for gastrointestinal tumors.^43^ Therefore, 600 J/cm^2^, which resulted in an ablation depth close to 5 mm, was chosen as the optimal irradiation energy for the in vivo study. By predicting the ablation range, PDT using the Ce6‐embedded stent‐based catheter system may provide an appropriate ablation range and reduce PDT‐related complications, such as colonic perforation.

### 2.5 Procedural outcomes of localized PDT in the rat colon

Endoluminal PDT using the Ce6‐embedded stent‐based catheter was technically successful in all rats without procedure‐related complications. All the stents fixed to the catheter were successfully placed, expanded, and removed under fluoroscopic guidance in a rat colon model (Figure S4). Local PDT using the Ce6‐embedded stent‐based catheter system can be performed with a single device from stent location to laser irradiation and can be removed immediately after the PDT procedure. This procedure eliminated stent‐related and PS‐related complications. The retrievability of the stent‐based catheter system seems to increase the safety of the local PDT procedure compared with previous studies of PDT therapy via stenting or intravenous PS injection.^29^, ^44^ All rats survived until the end of the study. Although the body weights of the PDT‐treated rats decreased slightly, this change did not significantly affect their general condition, behavior, and amount of food intake. The body weights gradually increased until their sacrifice (Figure S5). Colonic perforation or severe hemorrhage was not observed in any PDT‐treated rats. Although the ideal diameter of the stent for a rat colon model are not known, 2 mm larger than the original luminal diameter has been recommended as guidelines for colonic stents.^45^ In this study, the Ce6‐embedded stent with a diameter of 10 mm was used for the rat colon by considering the diameter of the water‐filled colon (8.49 ± 0.29 mm).^46^ The Ce6‐embedded stent‐based catheter was able to maximize the effects of PDT while maintaining sufficient contact with the inner wall of the colon. However, PDT using the Ce6‐embedded stent‐based catheter was performed in a normal rat colon. Therefore, endoluminal PDT using the Ce6‐embedded stent‐based catheter in an animal model with malignant colorectal obstruction should be investigated to verify its efficacy and safety.

### 2.6 Colonoscopic findings

**FIGURE 4:**
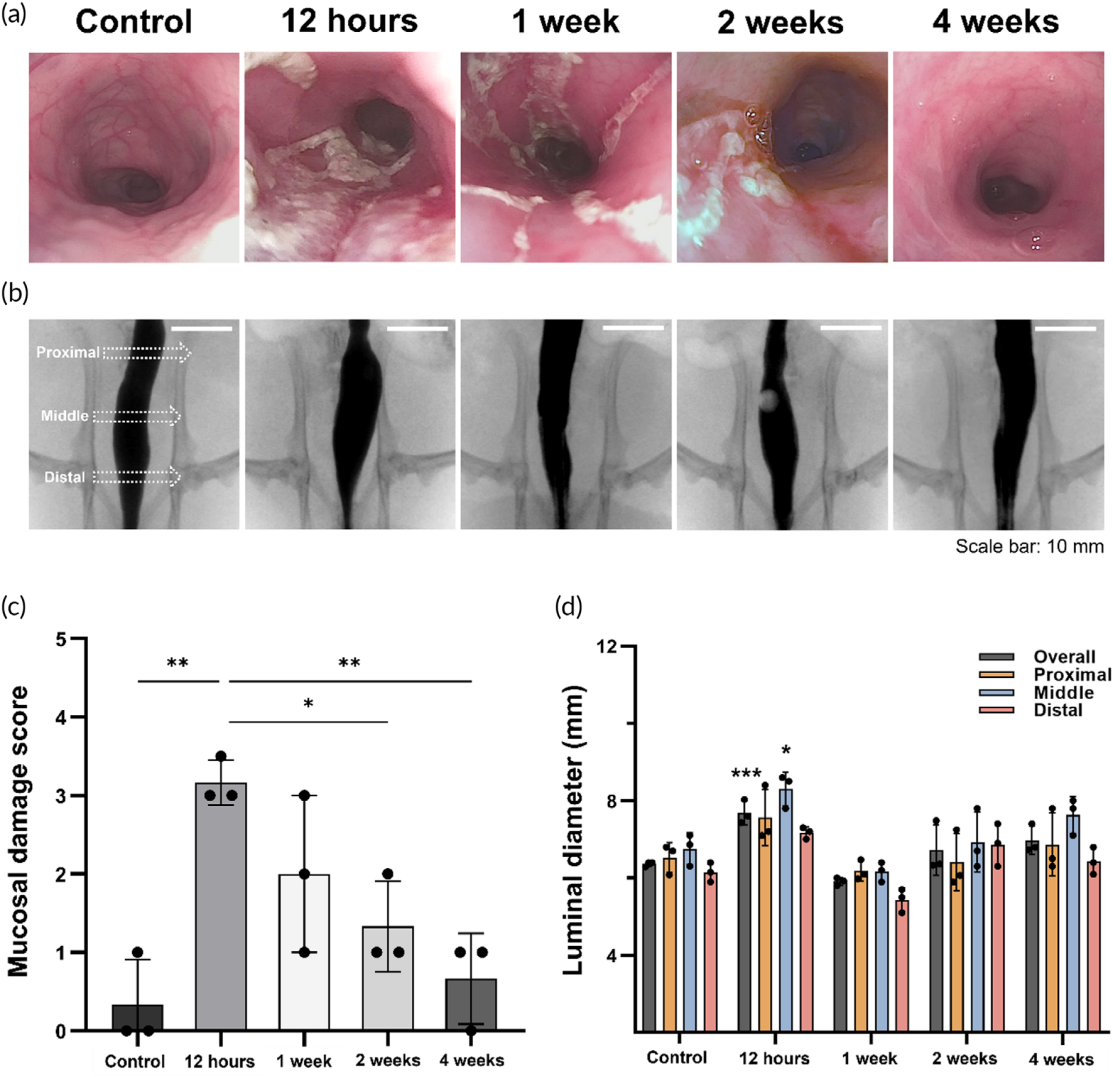
Follow‐up colonoscopic and colonographic findings after localized PDT procedure using Ce6‐embedded stent‐based catheter system in the rat colon model. (a) Representative photographs of the colonoscopic and (b) colonographic (scale bars: 10 mm) images in the control group and PDT‐treated groups at 12 h, 1, 2, and 4 weeks. (c) Graph showing the results of mucosal damage score after PDT. (d) Luminal diameter changes in the study groups. Data are presented as mean ± standard deviation. * *p* < 0.05, ** *p* < 0.01, *** *p* < 0.001. Ce6, Chlorin‐e6; PDT, photodynamic therapy.

Follow‐up colonoscopy and colonography were successfully performed at 12 h, 1, 2, and 4 weeks after PDT. The colonoscopic findings are shown in Figure 4a. Mucosal injuries with a white pseudomembrane were observed in the PDT‐treated colon at 12 h after PDT. These findings gradually recovered over 1–4 weeks. Follow‐up endoscopic examination revealed white mucosal damage at 2 days and 2 weeks after the procedure in a clinical trial.^47^ Such clinical data is consistent with our in vivo colonoscopic findings. Therefore, localized endoluminal PDT was successfully performed with the Ce6‐embedded stent‐based catheter. The mean (±SD) degree of mucosal damage was 0.33 ± 0.47 in the control group, 3.16 ± 0.23 at 12 h, 2 ± 0.81 at 1 week, 1.33 ± 0.47 at 2 weeks, and 0.66 ± 0.47 at 4 weeks after PDT, respectively (all variables, *p* < 0.01, one‐way ANOVA). The mean degree of mucosal damage was significantly higher at 12 h compared to that at the control (*p* < 0.01), 2 weeks (*p* < 0.05), and 4 weeks (*p* < 0.01). However, there was no statistical difference between 12 h and 1 week (*p* = 0.195) (Figure 4c). PDT with the PS‐embedded silicone membrane stimulated the mucosal of the colon; however, this effect was maintained for 2 weeks after the PDT procedure. Injuries to the mucosal tissues gradually recovered over time. Mucosal regeneration after single PDT procedure suggests a need for additional research on repeated and periodic PDT to maintain therapeutic effects. Clinical and pre‐clinical findings have demonstrated that repeated PDT effectively improves therapeutic outcomes of PDT procedures.^48^, ^49^, ^50^, ^51^, ^52^ In the previous localized PDT study, repeated PDT significantly increased cellular apoptosis and mucosal injuries compared to a single PDT procedure at 4 weeks follow‐up in a porcine model.^53^ Thus, repeated and periodic PDT using a photoreactive stent‐based catheter system to treat endoluminal malignancies may be effective for maintenance of circumferential damages of the non‐vascular luminal organs. Further investigation should be required to confirm the efficacy and safety of repeated PDT procedures.

### 2.7 Colonographic findings

The colonographic findings are shown in Figure 4b. The mean overall diameter significantly differed between the groups (all variables, *p* < 0.01, one‐way ANOVA). The mean (±SD) overall diameter in the PDT‐treated rats was significantly larger at 12 h (7.67 ± 0.26 mm, *p* < 0.001), but did not differ at 1 week (5.91 ± 0.08 mm, *p* = 0.298), 2 weeks (6.72 ± 0.53 mm, *p* = 0.884), and 4 weeks (6.97 ± 0.29 mm, *p* = 0.388), compared with that of the control group (6.47 ± 0.16 mm). The mean (±SD) diameter of the middle portion at 12 h (8.3 ± 0.35 mm, *p* < 0.05) was significantly higher than that in the control (6.73 ± 0.32 mm). However, the diameter slightly decreased at 1 week (6.16 ± 0.20, *p* = 0.620) and gradually recovered on 2 weeks (6.93 ± 0.63 mm, *p* = 0.991) and 4 weeks (7.63 ± 0.38 mm, *p* = 0.270) (Figure 4d). PDT with intravenous administration of PS was investigated for palliation in patients with esophageal cancer in clinical trials.^54^ Severe PDT‐related complications have been reported including the toxicity of systemic injected PS, esophageal perforation at the laser irradiation site, hemorrhage, and occurrence of esophageal strictures. Furthermore, all patients were instructed to avoid direct light exposure and stay in a room maintained at 500 lx or less for 2 weeks following the PDT. Our present findings revealed that the Ce6‐embedded stent‐based catheter system with laser irradiation successfully induced mucosal damage to the rat colon. The novel PS‐embedded stent‐based catheter system has several advantages compared with the previous studies. First, cylindrical structure of the stent‐based catheter system is suitable for localized ablation of the gastrointestinal tract. Second, its self‐expanding properties and high flexibility may be very useful for irregular or curved lesions of GI tract. Third, retrievability of the device immediately after PDT procedure can eliminate PS elution‐related toxicity. Also, stent‐related complication can be significantly reduced by eliminating the indwell time of PS‐embedded stent. This procedure was easy and safe therapeutic strategy under endoscopic or fluoroscopic guidance. This minimally‐invasive therapeutic strategy seems to be promising modality for local PDT treatment in malignant colorectal obstruction and could be expanded in non‐vascular luminal organs such as the esophagus, gastroduodenum, bile duct, urethra, and tracheal bronchus.

### 2.8 Histological findings

**FIGURE 5:**
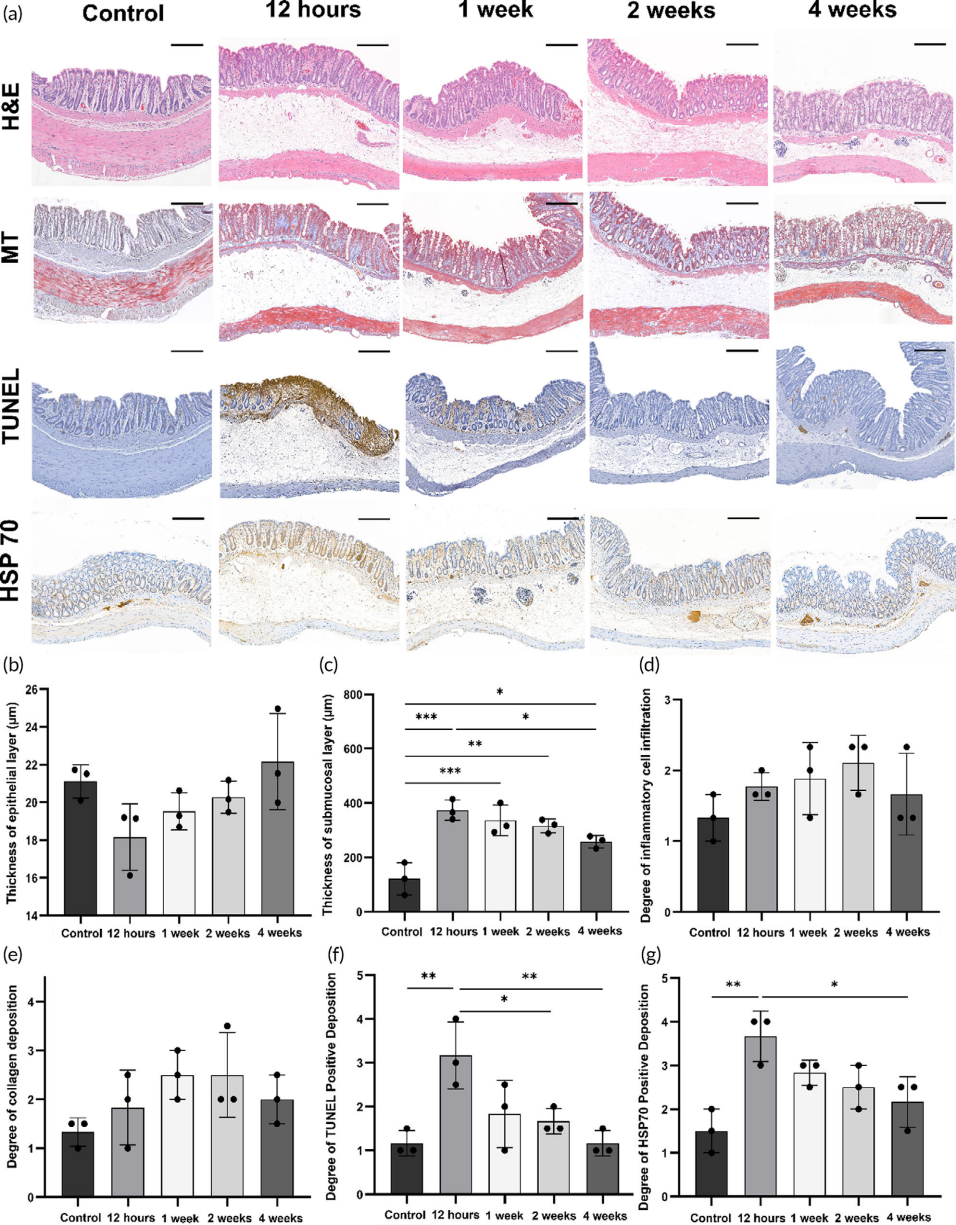
Histopathological findings in control group and PDT‐treated groups with Ce6‐embedded stent‐based catheter in the rat colon. (a) Representative microscopic images of hematoxylin & eosin‐, Masson's trichrome‐, HSP70‐, and TUNEL‐stained tissue slides (scale bars: 200 μm). (b)–(g) Histological results of the control and PDT‐treated groups regarding the thickness of the epithelial, submucosal layer, degree of inflammatory cell infiltration, collagen, TUNEL, and HSP70‐positive deposition. Data are presented as mean ± standard deviation. * *p* < 0.05, ** *p* < 0.01, *** *p* < 0.001. Ce6, Chlorin‐e6; HSP70, heat shock protein 70; PDT, Photodynamic therapy; TUNEL, terminal deoxynucleotidyl transferase‐mediated dUTP nick and labeling.

The entire colon of the rats was successfully extracted at the endpoint of the study. The histologic findings are summarized in Table S1, and their representative images are shown in Figure 5. The mean thickness of the submucosal layer and the degrees of terminal deoxynucleotidyl transferase‐mediated dUTP nick and labeling (TUNEL)‐positive deposition and heat shock protein (HSP70)‐positive deposition differed significantly between the groups (all variables *p* < 0.05, one‐way ANOVA). The mean thickness of the epithelial layer did not differ between the groups (*p* = 0.08); however, the thickness of the epithelial layer decreased immediately after PDT and gradually recovered over time. The mean thickness of the submucosal layer was significantly increased at 12 h (*p* < 0.001), 1 week (*p* < 0.001), 2 weeks (*p* = 0.01), and 4 weeks (*p* < 0.05) compared with that of the control. The degree of inflammatory cell infiltration (*p* = 0.305) and collagen deposition (*p* = 0.189) did not differ between the groups. The mean degree of TUNEL‐positive deposition was significantly higher at 12 h compared to that at the control (*p* < 0.01), 1 week (*p* = 0.071), 2 weeks (*p* < 0.05), and 4 weeks (*p* < 0.01). Similarly, the mean degree of HSP70‐positive deposition was also higher at 12 h compared with that in the control (*p* < 0.01) and those at 1 week (*p* = 0.314), 2 weeks (*p* = 0.098), and 4 weeks (*p* < 0.05). Previous studies that evaluated stent‐based RFA or IRE as localized ablation modalities in rat esophagus reported that the thickness of the epithelial layer had significantly decreased immediately after the procedure in the test group compared with the control group.^10^, ^12^ In contrast, PDT using the Ce6‐embedded stent‐based catheter demonstrated that the epithelial layer became slightly thinner without a statistically significant difference. Therefore, PDT using the PS‐embedded stent‐based catheter is a safer ablation method than stent‐based RFA and IRE in the colorectum, which is vulnerable to perforation. The effects of PDT efficacy is accompanied by the presence of edema, followed by the necrotic change in clinical or pre‐clinical situations.^54^, ^55^ Additionally, previous studies have reported a significantly higher positive rate of TUNEL‐staining in the PDT‐treated group after 12–24 h PDT.^56^, ^57^ Hereby, submucosal edema and apoptotic cell death within 12 h might be explained by the typical effects of PDT. In this study, HSP70 was overexpressed by 12 h. Subsequently, the degree of inflammatory cell infiltration and collagen deposition also gradually increased. In PDT‐induced apoptosis, cytoplasmic HSP70 is translocated to the cell surface, which initiates cell regeneration and wound healing, thus leading ultimately to collagen remodeling.^57^, ^58^, ^59^ Such a gradual regeneration process after PDT is consistent with our in vivo findings and clinical data of PDT in patients with esophageal cancer.^60^, ^61^ Therefore, PDT using the Ce6‐embedded stent‐based catheter was successful and demonstrated the typical features of PDT process, from apoptosis or necrosis to recovery.

## 3 CONCLUSIONS

A photoreactive stent‐based catheter system was successfully developed as a novel PDT platform to improve the therapeutic effects in endoluminal organs. A Ce6‐embedded stent was suitable for the push‐pull design of the catheter, which was demonstrated by the stabilization ability of the mPEG‐Ce6‐embedded silicone membrane after deployment. The Ce6‐embedded pieces were photodynamically active against colon cancer cells and the phototoxicity was proportionally increased to the irradiation energy. Three different irradiation energy doses (200, 400, and 600 J/cm^2^) were used to investigate the dose–range effects; a dose of 600 J/cm^2^, which resulted in sufficient ablation depth and even ablation, was chosen as the optimal irradiation energy in the in vivo study. Endoluminal PDT using the photoreactive stent‐based catheter system with even and accurate delivery of light source was successfully performed in a rat colon model. The results of colonoscopy, colonography, histology, and statistical analyses demonstrated that endoluminal PDT using the Ce6‐embedded stent‐based catheter was effective and safe to evenly induce mucosal injuries in the rat colon. Although additional studies are required to further validate the current findings, the photoreactive stent‐based catheter system produced localized PDT‐induced therapeutic effects by fully contacting the inner walls of the rat colon, while eliminating the current PS‐elution and stent‐related complications. Therefore, endoluminal PDT using the Ce6‐embedded stent‐based catheter system may represent a promising novel approach for treating endoluminal malignancies in non‐vascular organs.

## 4 EXPERIMENTAL SECTION

### 4.1 Materials

Ce6 was purchased from Frontier Scientific, Inc. (Logan, UT, USA). mPEG‐AM (molecular weight; 5 kDa) was purchased from Sun Bio (Gunpo‐si, Gyeonggi‐do, Korea). Tetrahydrofuran and xylene were purchased from Junsei Co. (Tokyo, Japan), and TTC was purchased from Sigma–Aldrich Co. (St. Louis, MO, USA). Silicone derivatives (MED‐6640) were purchased from Nusil™ Technology LLC. (Carpinteria, CA, USA). SOSG (Cat. no. S36002) was purchased from Thermo Fisher Scientific (Waltham, MA, USA). Dulbecco modified eagle medium (DMEM, Cat. no. SH30243.01), and fetal bovine serum (FBS, Cat. no. SH30919.03) were purchased from GE Healthcare Life Sciences (Logan, UT, USA). Penicillin–streptomycin (Cat. no. 17‐745E) was purchased from Lonza Bioscience (Walkersville, MD, USA). CCK‐8 kit (Cat. no. CCK‐3000) was purchased from DOJINDO (Kumamoto, Japan). Calcein AM/EthD‐1 LIVE/DEAD® Viability/Cytotoxicity kit (Cat. no. L3224) was purchased from Invitrogen (Carlsbad, CA, USA). CT‐26 cell line was purchased from Korean Cell Line Bank (Seoul, Korea). Porcine livers were purchased from Biozoa (Seoul, Korea). The cylindrical fiber was purchased from Biolitec MILON Group (London, UK). The laser system (LAB671‐1000MWCW400F) was purchased from JM Labtech (Seoul, Korea). The Ce6‐embedded piece, stent, and stent‐based catheter system were supplied by S&G Biotech (Yongin, Gyeonggi‐do, Korea).

### 4.2 Preparation of Ce6‐embedded stent‐based catheter samples

The SEMS was fabricated using a hand‐knitted technique with a single thread of 0.127‐mm‐thick Ni—Ti alloy wire into a tubular diamond‐cell configuration. The preparation of synthesized mPEG‐Ce6‐silicone and fabrication of the Ce6‐embedded stent are the same as in the previously described.^28^, ^29^ In brief, the fabricated stent was dipped in the Ce6‐silicone coating solution for 10 s followed by slow withdrawal. The stent was immediately dried in an oven at 150°C for 3 h and air‐dried for 30 min at room temperature. The Ce6‐embedded pieces for the in vitro cell study were 1 cm × 1 cm (1 cm^2^). The Ce6‐embedded stents with 5 mm in diameter and 10 mm in length were used for the dose–range study in porcine liver. The Ce6‐embedded stent‐based catheters with 10 mm in diameter and 60 mm in length stent were used in the in vivo study. For the Ce6‐embedded stent‐based catheter fabrication, both ends of the stent were fixed to the catheter system for recapture and removal immediately after PDT. The delivery system was 10‐Fr in diameter and 40 cm in usable length and comprised an insulating outer sheath and a pusher catheter with a guiding olive tip. A cylindrical fiber could be inserted through the distal port of the catheter and advanced along the inner pathway until it reached the center of the stent to perform accurate PDT to the target region.

### 4.3 Characterization of mPEG‐Ce6 stent‐based catheter system

The compound of the stent membrane was confirmed by FT‐IR. The singlet oxygen generation efficacy of the stents was measured using SOSG. To measure the singlet oxygen generation, each stent was placed in a SOSG solution. The mPEG‐Ce6 stent was irradiated with 670 nm red light (Fiber Coupled Laser Modules, LaserLab®, Seoul, Korea) (total 10 J/cm^2^, 20 s, 25 measurements). The fluorescence intensity of SOSG was detected using fluorescence spectroscopy (RF‐5301, Shimadzu, Kyoto, Japan) (*E*x/*E*m = 504 nm/525 nm). Photo‐fluorescence imaging with Image Station 4000 MM (Kodak, New Haven, CT) was observed to evaluate the photodynamic activity of before loading and after deployment of mPEG‐Ce6 stent, and the results were compared with those of a silicone stent.

### 4.4 Mechanical properties of mPEG‐Ce6 stent‐based catheter system

The radial and axial forces of the fully expanded stent portion of the stent‐based catheter system were investigated to evaluate the expansion performance and flexibility. The radial force was measured using a radial force testing machine (TTR2, Blockwise Engineering, Tempe, AZ, USA). 5 min before testing, the sample was put on the crimping head. The tester program was designed to reduce the stent diameter from the unconstrained 10 mm to a predetermined 5 mm. The axial force was measured using a universal testing machine (LR30KPlus, Lloyd Instruments Ltd., Bognor Regis, UK). A stent‐based catheter was positioned on the bottom plate of the machine, and the upper plate was moved to axially reduce the stent diameter from 10 mm to a predetermined 5 mm. All measurements were performed at a speed of 0.5 mm/s. Forces and displacement values were recorded at intervals of 0.05 s on force–diameter and force–displacement graphs. The test was performed under the load‐controlled feedback mode with a 20 s linear loading at a peak force of 50 kgf.

### 4.5 PDT using the Ce6‐embedded piece on CT‐26 cells

The CT‐26 cells were cultured in DMEM containing 10% FBS and 1% penicillin/streptomycin in 5% CO_2_ at 37°C. The cells were seeded in 12‐well plates at a density of 5 × 10^5^ cells per well and incubated for 24 h. Of the 12 well, nine were subjected for PDT (irradiation energy: 1, 3, or 5 J/cm^2^, *n* = 3 in each group), and the remaining three wells were used as the sham control for presenting a normal cell by placing the Ce6‐embedded piece for 100 s without laser irradiation. Subsequently, 24 h after seeding, the media was removed, and the Ce6‐embedded pieces were placed on the cell monolayer in each well. Laser irradiation was performed on a customized blackboard to minimize the interference between different wells. After irradiation, 1 mL of fresh medium was added, and the piece was removed.

### 4.6 Cell viability

In vitro cell viability after the laser irradiation was qualitatively analyzed using the Calcein AM/EthD‐1 LIVE/DEAD® Viability/Cytotoxicity Kit and the D‐Plus^TM^ CCK cell viability assay Kit according to the manufacturer's instructions. To perform the live/dead assay, after the laser irradiation, 500 μL of Calcein AM and EthD‐1 mixed solution was added to each well and incubated for 30 min at room temperature. Live cells were stained with Calcein AM, whereas dead cells were stained with EthD‐1. The stained cells were imaged using a fluorescence microscope (EVOS FL Auto, Life Technologies, Carlsbad, USA), and ImageJ 1.53c (National Institutes of Health, Bethesda, MD) was used to analyze the fluorescence imaging results. To measure cell viability, the each well was added 500 μL of a 10% CCK‐8 solution, and the cells were incubated for another 4 h. Next, 100 μL from each resulting sample were transferred to 96‐well plates, and the absorbance at 450 nm was determined using a microplate reader (Biotek Instruments Inc., Winooski, USA) and analyzed with Gen5 software (Agilent Technologies, Santa Clara, CA). Cell viability was expressed as a percentage compared with the control.

### 4.7 PDT dose–range study in porcine liver

The porcine liver was sectioned into nine cuboids (height: 35 mm, length: 40 mm, width: 40 mm) to determine the optimal irradiation energy. A cylindrical hole was acquired using a steel wire with an outer diameter of 1.6 mm. The Ce6‐embedded stent‐loaded delivery system was inserted through the hole. The stent was fully expanded in the middle portion of the cuboid‐shaped liver under fluoroscopic guidance (MeteoR, NanoFocusRay Co., Iksan, Korea). A cylindrical fiber (irradiation length: 2 cm) was carefully inserted in the center portion of the placed stent. PDT was performed with three irradiation energies of 200, 400, and 600 J/cm^2^. The chosen doses are within the typical oncological range of 100–600 J/cm^2^.^42^ Laser irradiation with a wavelength of 670 nm was administered at a power density of 1000 mW/cm^2^ using a laser system. The PDT‐treated livers were transversely sectioned, and TTC was used to evaluate cell viability. Images of the liver slices were obtained 12 h later, and the PDT‐induced ablation depth was measured using ImageJ 1.53c software. The ablation depth was defined as the maximum depth of ablated tissue on a transverse section.

### 4.8 Animal study design

This study was approved by the Institutional Animal Care and Use Committee (IACUC approval number 2022‐12‐159, Asan Medical Center, Seoul, Korea) and conformed to the US National Institutes of Health guidelines for the humane handling of laboratory animals. A total of 15 Sprague–Dawley rats weighing 332–420.5 g (males; mean weight, 378.7 g; JA BIO, Seoul, Korea) were used. The experiment was performed with males to minimize variability due to the menstrual cycle.^62^ 12 of 15 rats underwent PDT with the Ce6‐embedded stent‐based catheter system and were randomly selected to be sacrificed at 12 h (*n* = 3), 1 week (*n* = 3), 2 weeks (*n* = 3), and 4 weeks (*n* = 3) after the procedure by administering inhalable pure carbon dioxide. The remaining three rats were used as the control for presenting with a normal colon without PDT. All animals were housed under the same environmental conditions (temperature of 24 ± 2°C with a 12 h day‐night cycle) and were supplied with water and food ad libitum.

### 4.9 PDT using the Ce6‐embedded stent‐based catheter system in rat colon

After a 24‐h fasting period with free access to drinking water, anesthesia was induced with an intramuscular injection of 50 mg/kg zolazepam, 50 mg/kg tiletamine (Zoletil 50; Virbac, Carros, France), and 10 mg/kg xylazine (Rompun; Bayer HealthCare, Leverkusen, Germany). Enema was performed by inserting a 6‐Fr sheath. A pre‐procedural colon study was obtained to determine the position of the rat colon. A 0.035‐inch guidewire (Radifocus M; Terumo, Tokyo, Japan) was inserted through the anus and advanced to the distal colon. The Ce6‐embedded stent‐based catheter was inserted over the guidewire into the colon under fluoroscopic guidance (Figure S4a). The distal end of the Ce6‐embedded stent was located 15 mm from the anal verge. The Ce6‐embedded stent fixed to the catheter was deployed by smoothly pulling the braided tube to pusher catheter (Figure S4b). The guidewire was removed with the Ce6‐embedded stent‐based catheter left in place. The cylindrical fiber was inserted into the middle portion of the Ce6‐embedded stent through the distal port of the catheter (Figure S4c). Laser irradiation (wavelength, 670 nm) was performed at a power density of 1000 mW/cm^2^ and irradiation energy of 600 J/cm^2^ using a laser system. After PDT, the cylindrical fiber was removed. Subsequently, the expanded Ce6‐embedded stent was recaptured by advancing the braided tube, and the catheter was smoothly removed (Figure S4d). A post‐procedural contrast study was performed to confirm the presence of PDT‐related colonic perforation. Antibiotics (gentamicin, 40 mg/mL; Shin Poong Pharm Ltd., Seoul, Korea) and analgesics (keromin, 30 mg; Ketorolac; Hana Pharm Ltd., Seoul, Korea) were routinely used for 3 days after the procedure.

### 4.10 Follow‐up colonoscopy and colonography

Follow‐up endoscopic examination and contrast study were performed before and immediately after PDT and before sacrifice at 12 h, and 1, 2, and 4 weeks in all rats. Endoscopic examination using a CMOS Video‐Rhino‐Layngoscope (Karl Storz, Tuttlingen, Germany) was performed to evaluate the PDT‐treated mucosal changes of the rat colon. The degree of the mucosal damage was assessed with mucosal damage score of the Modified Rachmilewitz Endoscopic Index as follows: none (0), slight (2), or pronounced (4).^63^ Colonography was performed using a contrast medium (Telebrix Gastro; Guerbet, Villepinte, France) to evaluate the luminal patency of the PDT‐treated colon. The colonic luminal diameters were measured in all rats at the three segments of the PDT‐treated colon using ImageJ software.

### 4.11 Histological examinations

Surgical exploration of the entire colon was performed in all rats. The extracted tissue samples were immediately fixed in 10% neutral buffered formalin for 24 h. The PDT‐treated colon was transversely sectioned. The samples were stained with hematoxylin & eosin (H&E) and Masson's trichrome (MT). The H&E‐stained sections were used to assess the thicknesses of the epithelial and submucosal layers, averaging measurements at eight points around the circumference. Inflammatory cell infiltration was subjectively determined in accordance with the distribution and density of the inflammatory cells (graded as 1, mild; 2, mild‐to‐moderate; 3, moderate; 4, moderate‐to‐severe; and 5, severe). The degree of collagen deposition was subjectively determined using MT‐stained sections (grading: 1, mild; 2, mild‐to‐moderate; 3, moderate; 4, moderate‐to‐severe; and 5, severe). Histological analyses were performed with a digital slide scanner (Panoramic 250 FLASH III; 3D Histech Ltd., Budapest, Hungary) and measurements were obtained with a digital microscope viewer (CaseViewer; 3D Histech). Histological analysis was performed by a consensus of three observers who were blinded to the animal groups.

### 4.12 Immunohistochemistry

Immunohistochemistry (IHC) was performed to evaluate the extent of cell death and the presence of translocated cytoplasmic HSP70 (LS‐B3700‐50; LifeSpan BioSciences Inc., Seattle, WA, USA). IHC was performed on paraffin‐embedded tissue sections using TUNEL (ApopTag Peroxidase In Situ Detection kit; Millipore Co., Burlington, MA, USA) and HSP70 primary antibodies. The degrees of TUNEL and HSP70‐positive depositions were subjectively determined (1, mild; 2, mild‐to‐moderate; 3, moderate; 4, moderate‐to‐severe; and 5, severe). IHC analyses were performed similar to the histological examinations.

### 4.13 Statistical analysis

Data are expressed as mean ± standard deviation. Differences between the groups were analyzed using the two‐sample *t*‐test or one‐way ANOVA, and Tukey's method was used as the post hoc test for ANOVA, as appropriate. *p*‐values <0.05 were considered statistically significant. Statistical analyses were performed using Prism (GraphPad version 5.0.0; La Jolla, CA, USA) and SPSS v27.0 (IBM, Chicago, IL, USA). Significance was noted as follows: * *p* < 0.05, ** *p* < 0.01, *** *p* < 0.001.

## AUTHOR CONTRIBUTIONS

**Seung Jin Eo:** Investigation; validation; writing – original draft. **Dae Sung Ryu:** Investigation; writing – original draft; funding acquisition. **Hyeonseung Lee:** Investigation; validation; writing – original draft. **Ji Won Kim:** Methodology; validation. **Song Hee Kim:** Methodology; validation. **Jin Hee Noh:** Methodology; writing – review and editing. **Yuri Kim:** Methodology; writing – review and editing. **Seokin Kang:** Methodology; writing – review and editing. **Kun Na:** Conceptualization; supervision; resources; writing – review and editing. **Jung‐Hoon Park:** Conceptualization; supervision; resources; writing – review and editing; funding acquisition. **Do Hoon Kim:** Conceptualization; funding acquisition; writing – review and editing.

## CONFLICT OF INTEREST STATEMENT

The authors have no conflicts of interest to declare.

## Supporting information


**FIGURE S1.** Photographs show the Ce6‐embedded stent‐based catheter system to perform endoluminal PDT. (a) The Ce6‐embedded stent is loaded into the catheter system. (b) The stent is deployed by pulling the braided tube (*arrowheads*). (c) After full deployment, a cylindrical fiber (*white arrows*) was inserted into the middle portion of the Ce6‐embedded stent. (d) PDT was administered using laser irradiation. Ce6, Chlorin‐e6; PDT, photodynamic therapy.
**FIGURE S2.** Photograph and a schematic illustration of the *in vitro* study. Ce6‐embedded pieces were placed on the cell monolayer and irradiated using the laser. Ce6, Chlorin‐e6.
**FIGURE S3.** PDT using Ce6‐embedded stent in the porcine liver tissue. The ablation depth was defined as the maximum length ablated along the transverse section in TTC‐stained liver tissues. PDT, Photodynamic therapy; Ce6, Chlorin‐e6; TTC, 2,3,5‐triphenyltetrazolium chloride.
**FIGURE S4.** Radiographic images showing the technical steps for localized PDT using Ce6‐embedded stent‐based catheter system in the rat colon. (a) A 0.035‐inch guidewire was advanced through the anus into the distal colon, and the Ce6‐embedded stent‐based catheter (arrowheads) was inserted over the guidewire. The distal end of the Ce6‐embedded stent (arrow) was placed 15 mm from the anal verge under fluoroscopic guidance. (b) The Ce6‐embedded stent (arrowheads) was deployed by pulling the braided tube (black arrow). (c) The cylindrical fiber (arrows) was inserted into the middle portion of the stent (arrowheads) through the distal port of the catheter to administer localized PDT. (d) After the PDT, the fiber was removed. Then, the expanded stent was recaptured by advancing the braided tube (black arrow), and the catheter system was smoothly removed. PDT, Photodynamic therapy; Ce6, Chlorin‐e6.
**FIGURE S5.** The body weight changes after PDT procedure in enrolled rats. PDT, photodynamic therapy.
**TABLE S1.** Histological findings in all groups of rats.

## Data Availability

The data that support the findings of this study are available from the corresponding author upon reasonable request.
