# Supplementary material for: Endoluminal photodynamic therapy with a photoreactive stent‐based catheter system to treat malignant colorectal obstruction
Source: Bioeng Transl Med. 2024 Nov 23;10(2):e10732. doi: 10.1002/btm2.10732 (PMC11883124; doi:10.1002/btm2.10732)
Supplement: Supplementary file 1 — FIGURE S1. Photographs show the Ce6‐embedded stent‐based catheter system to perform endoluminal PDT. (a) The Ce6‐embedded stent is loaded into the catheter system. (b) The stent is deployed by pulling the braided tube (arrowheads). (c) After full deployment, a cylindrical fiber (white arrows) was inserted into the middle portion of the Ce6‐embedded stent. (d) PDT was administered using laser irradiation. Ce6, Chlorin‐e6; PDT, photodynamic therapy. FIGURE S2. Photograph and a schematic illustration of the in vitro study. Ce6‐embedded pieces were placed on the cell monolayer and irradiated using the laser. Ce6, Chlorin‐e6. FIGURE S3. PDT using Ce6‐embedded stent in the porcine liver tissue. The ablation depth was defined as the maximum length ablated along the transverse section in TTC‐stained liver tissues. PDT, Photodynamic therapy; Ce6, Chlorin‐e6; TTC, 2,3,5‐triphenyltetrazolium chloride. FIGURE S4. Radiographic images showing the technical steps for localized PDT using Ce6‐embedded stent‐based catheter system in the rat colon. (a) A 0.035‐inch guidewire was advanced through the anus into the distal colon, and the Ce6‐embedded stent‐based catheter (arrowheads) was inserted over the guidewire. The distal end of the Ce6‐embedded stent (arrow) was placed 15 mm from the anal verge under fluoroscopic guidance. (b) The Ce6‐embedded stent (arrowheads) was deployed by pulling the braided tube (black arrow). (c) The cylindrical fiber (arrows) was inserted into the middle portion of the stent (arrowheads) through the distal port of the catheter to administer localized PDT. (d) After the PDT, the fiber was removed. Then, the expanded stent was recaptured by advancing the braided tube (black arrow), and the catheter system was smoothly removed. PDT, Photodynamic therapy; Ce6, Chlorin‐e6. FIGURE S5. The body weight changes after PDT procedure in enrolled rats. PDT, photodynamic therapy. TABLE S1. Histological findings in all groups of rats. [file BTM2-10-e10732-s001.docx]

**Endoluminal Photodynamic Therapy with a Photoreactive Stent-based Catheter System to Treat Malignant Colorectal Obstruction**

Seung Jin Eo ^a,b,1^, Dae Sung Ryu ^a,c,1^, Hyeonseung Lee ^d,1^ , Ji Won Kim ^a,c^, Song Hee Kim ^a,c^, Jin Hee Noh ^c^, Yuri Kim ^c^, Seokin Kang ^e^, Kun Na ^d,^*, Jung-Hoon Park ^a,b,^*, and Do Hoon Kim ^c,^*

^a^ Biomedical Engineering Research Center, Asan Institute for Life Sciences, Asan Medical Center, 88 Olympic-ro 43-gil, Songpa-gu, Seoul, 05505, Republic of Korea

^b^ Department of Convergence Medicine, Asan Medical Center, University of Ulsan College of Medicine, 88 Olympic-ro 43-gil, Songpa-gu, Seoul, 05505, Republic of Korea

^c^ Department of Gastroenterology, Asan Medical Center, University of Ulsan College of Medicine, 88 Olympic-ro 43-gil, Songpa-gu, Seoul, 05505, Republic of Korea

^d^ Department of Biotechnology, Department of Biomedical-Chemical Engineering, The Catholic University of Korea, 43 Jibong-ro, Wonmi-gu, Bucheon-si, Gyeonggi-do, 14662, Republic of Korea

^e^ Department of Internal Medicine, Ilsan Paik Hospital, Inje University College of Medicine, 170, Juhwa-ro, Ilsanseo-gu, Goyang, Gyeonggi-do, 10380, Republic of Korea

^1^ S.J.E., D.S.R., and H.L. contributed equally to this work and are the co-first authors.

^*^ K.N., J.-H.P., and D.H.K. contributed equally to this work and are the co-corresponding authors.


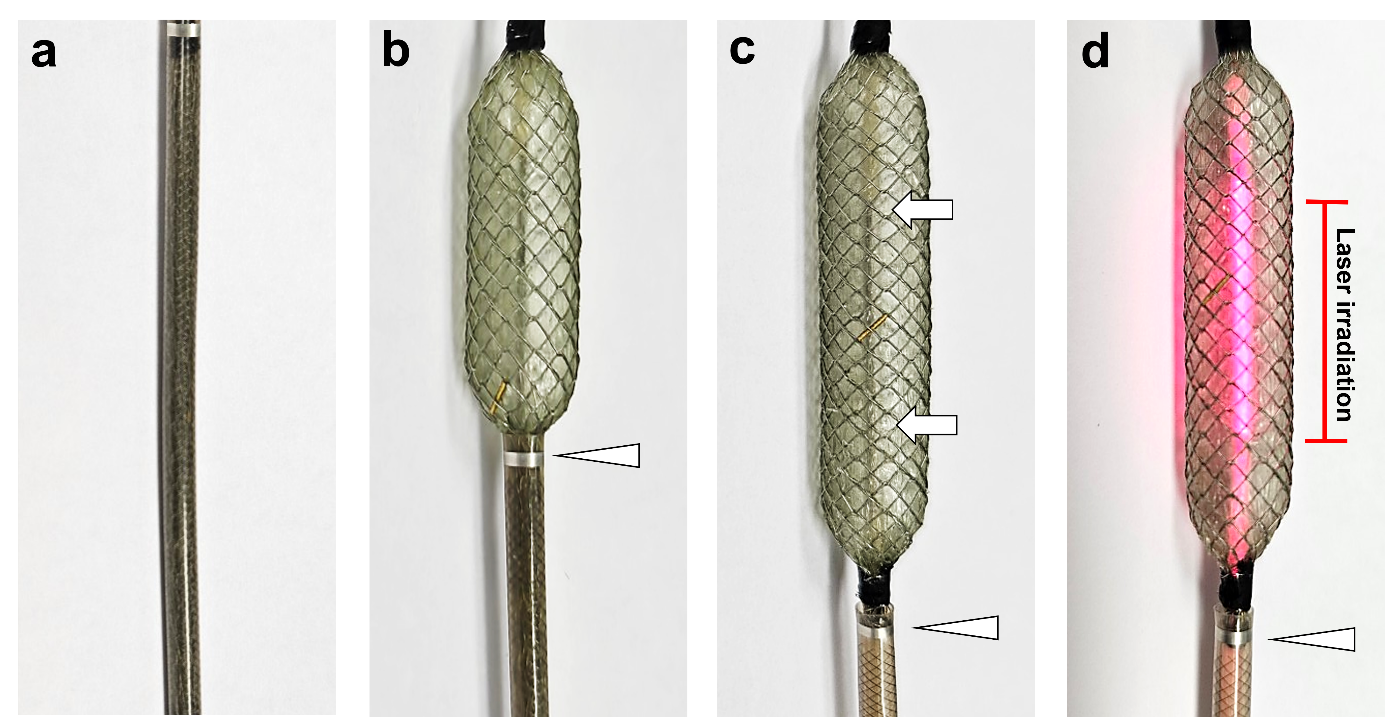
**Supplemental Figure 1.** Photographs show the Ce6-embedded stent-based catheter system to perform endoluminal PDT. a) The Ce6-embedded stent is loaded into the catheter system. b) The stent is deployed by pulling the braided tube (*arrowheads)*. c) After full deployment, a cylindrical fiber (*white arrows*) was inserted into the middle portion of the Ce6-embedded stent. d) PDT was administered using laser irradiation. Ce6, Chlorin-e6; PDT, photodynamic therapy.


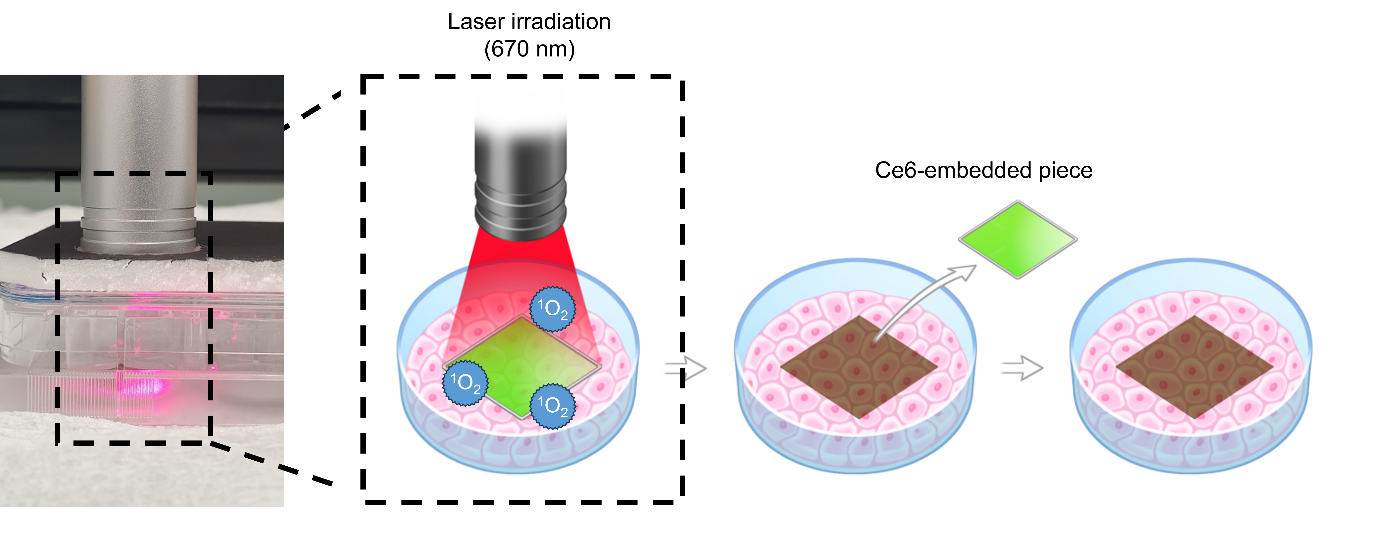


**Supplemental Figure 2.** Photograph and a schematic illustration of the *in vitro* study. Ce6-embedded pieces were placed on the cell monolayer and irradiated using the laser. Ce6, Chlorin-e6.


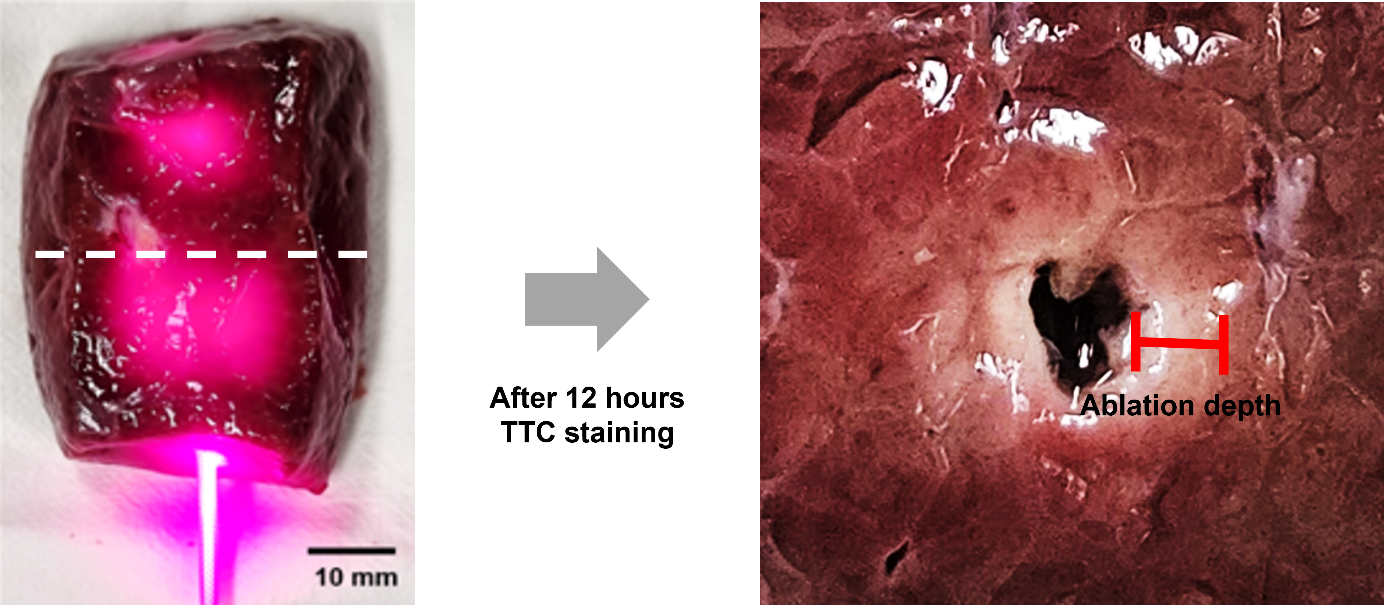


**Supplemental Figure 3.** PDT using Ce6-embedded stent in the porcine liver tissue. The ablation depth was defined as the maximum length ablated along the transverse section in TTC-stained liver tissues. PDT, Photodynamic therapy; Ce6, Chlorin-e6; TTC, 2,3,5-triphenyltetrazolium chloride.


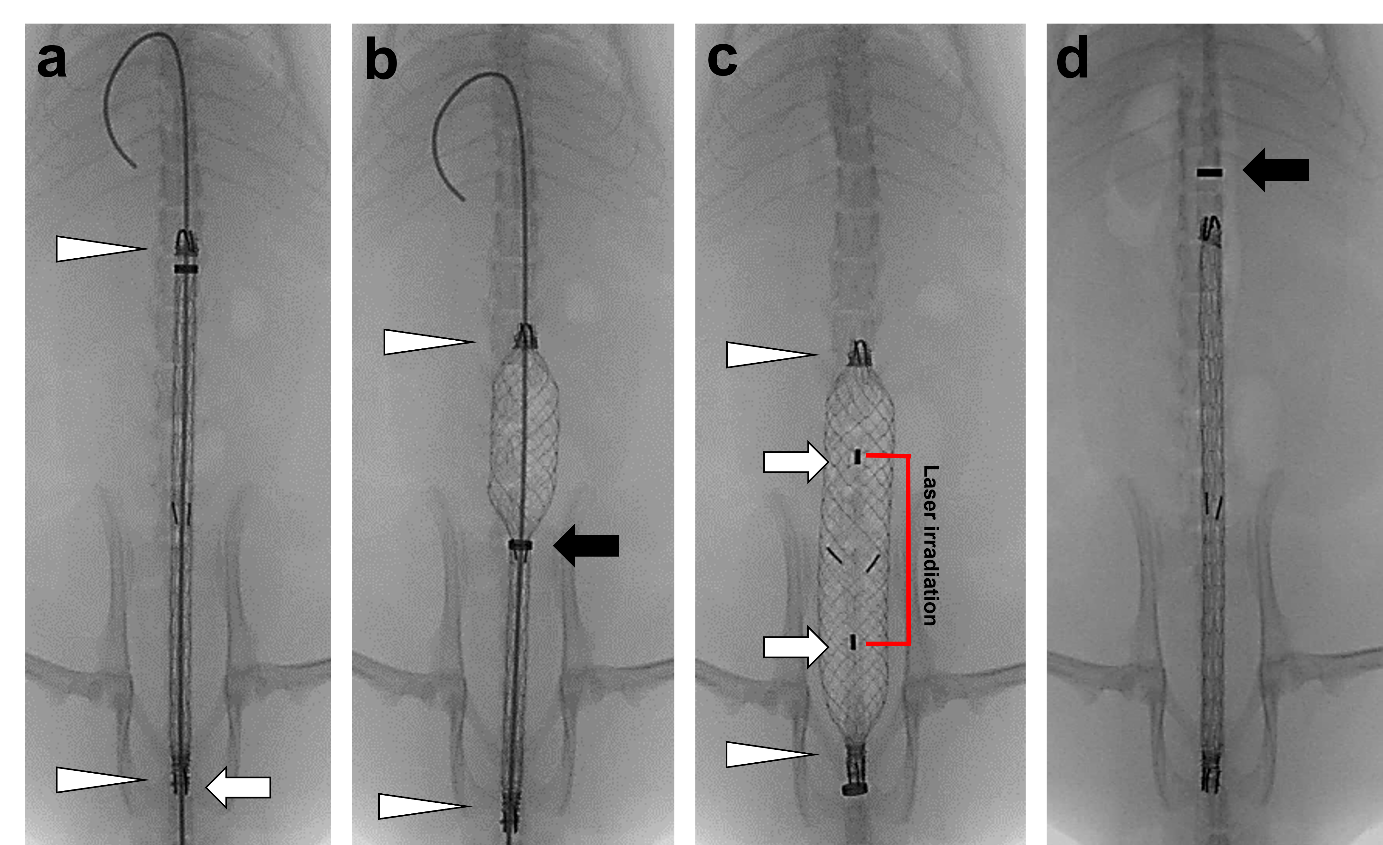


**Supplemental Figure 4.** Radiographic images showing the technical steps for localized PDT using Ce6-embedded stent-based catheter system in the rat colon. a) A 0.035-inch guidewire was advanced through the anus into the distal colon, and the Ce6-embedded stent-based catheter (arrowheads) was inserted over the guidewire. The distal end of the Ce6-embedded stent (arrow) was placed 15 mm from the anal verge under fluoroscopic guidance. b) The Ce6-embedded stent (arrowheads) was deployed by pulling the braided tube (black arrow). c) The cylindrical fiber (arrows) was inserted into the middle portion of the stent (arrowheads) through the distal port of the catheter to administer localized PDT. d) After the PDT, the fiber was removed. Then, the expanded stent was recaptured by advancing the braided tube (black arrow), and the catheter system was smoothly removed. PDT, Photodynamic therapy; Ce6, Chlorin-e6.


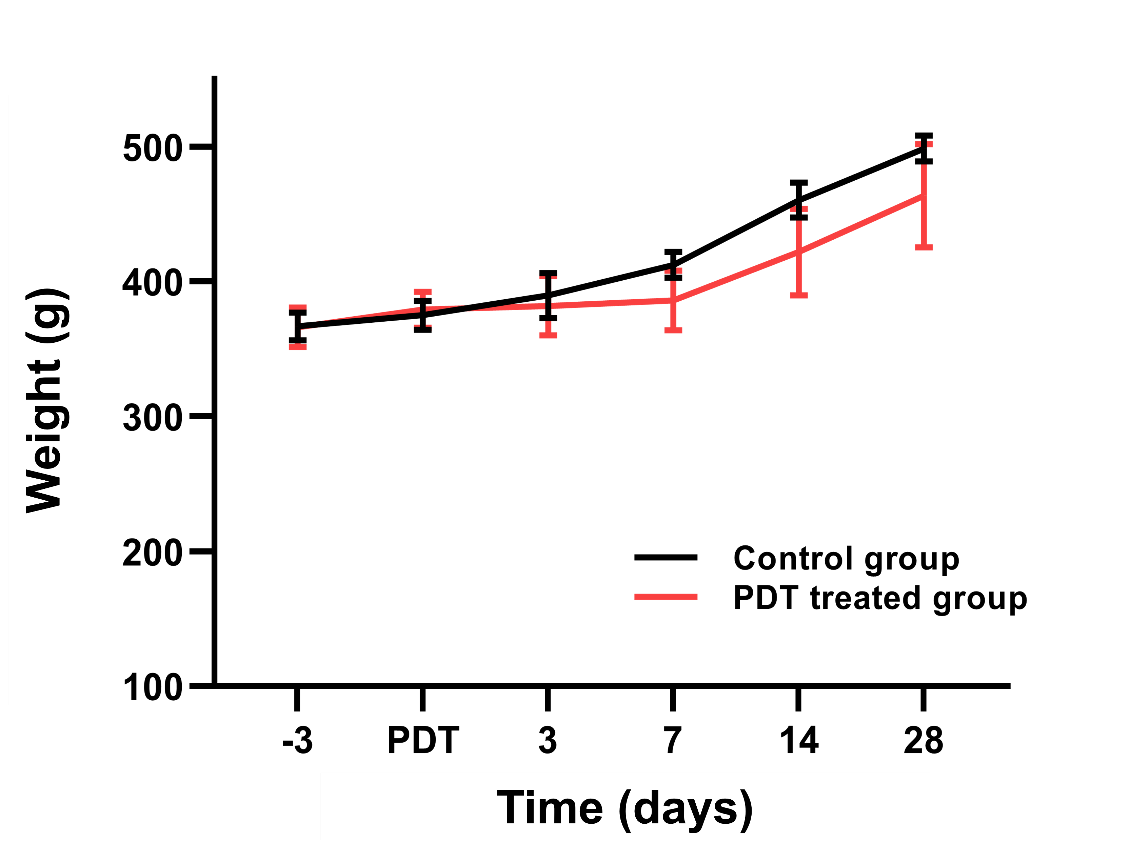


**Supplemental Figure 5.** The body weight changes after PDT procedure in enrolled rats. PDT, photodynamic therapy.

**Supplemental Table 1.** Histological findings in all groups of rats.

|  | **Groups** | | | | | ***p*-value*** | ***p*-value** | | | | | | | | | | |
| --- | --- | --- | --- | --- | --- | --- | --- | --- | --- | --- | --- | --- | --- | --- | --- | --- | --- |
|  | ^A^ Control | ^B^ 12 hours | ^C^ 1 week | ^D^ 2 weeks | ^E^ 4 weeks |  | A vs. B | A vs. C | A vs. D | A vs. E | B vs. C | B vs. D | B vs. E | C vs. D | C vs. E | D vs. E |  |
| Epithelial layer (μm) | 21.11 ± 0.72 | 18.16 ± 1.43 | 19.53 ± 0.80 | 20.27 ± 0.69 | 22.15 ± 2.09 | *0.080* | *0.213* | *0.728* | *0.960* | *0.917* | *0.811* | *0.494* | *0.062* | *0.975* | *0.303* | *0.591* |  |
| Submucosal layer (μm) | 134.54 ± 33.08 | 373.51 ± 30.58 | 336.35 ± 46.26 | 315.49 ± 20.84 | 257.16 ± 18.72 | *<0.001* | *<0.001* | *<0.001* | *0.01* | *0.02* | *0.762* | *0.402* | *0.027* | *0.960* | *0.163* | *0.398* |  |
| Inflammatory cell (degree) | 1.33 ± 0.27 | 1.77 ± 0.15 | 1.88 ± 0.41 | 2.11 ± 0.31 | 1.55 ± 0.56 | 0.305 | *0.705* | *0.520* | *0.236* | *0.864* | *0.997* | *0.864* | *0.997* | *0.965* | *0.963* | *0.705* |  |
| Collagen (degree) | 1.33 ± 0.23 | 1.83 ± 0.62 | 2.5 ±0.40 | 2.5 ±0.70 | 2 ± 0.40 | 0.189 | *0.854* | *0.219* | *0.219* | *0.687* | *0.687* | *0.411* | *0.997* | *1.000* | *0.854* | *0.854* |  |
| TUNEL (degree) | 1.16 ± 0.23 | 3.16 ± 0.62 | 1.83 ± 0.62 | 1.66 ± 0.23 | 1.16 ±0.23 | 0.006 | *0.007* | *0.566* | *0.778* | *1.000* | *0.071* | *0.039* | *0.007* | *0.995* | *0.566* | *0.778* |  |
| HSP 70 (degree) | 1.5 ± 0.40 | 3.66 ± 0.47 | 2.83 ± 0.23 | 2.5 ± 0.40 | 2.16 ± 0.47 | *0.004* | *0.002* | *0.052* | *0.179* | *0.511* | *0.314* | *0.098* | *0.028* | *0.920* | *0.511* | *0.920* |  |

Data are presented as mean ± standard deviations. *One-way ANOVA test. TUNEL: terminal deoxynucleotidyl transferase-mediated dUTP, HSP70: heat shock protein 70.
